# Quantitative design rules for protein-resistant surface coatings using machine learning

**DOI:** 10.1038/s41598-018-36597-5

**Published:** 2019-01-22

**Authors:** Tu C. Le, Matthew Penna, David A. Winkler, Irene Yarovsky

**Affiliations:** 1 School of Engineering, RMIT University, GPO Box 2476, Melbourne, Victoria, 3001 Australia; 2 ARC Industrial Transformation Research Hub for Australian Steel Manufacturing, Wollongong, NSW, 2522, Australia; 3 Monash Institute of Pharmaceutical Sciences, Monash University, Parkville, Victoria, 3052 Australia; 4 La Trobe Institute for Molecular Science, La Trobe University, Bundoora, Victoria, 3084 Australia; 5 CSIRO Manufacturing, Clayton, Victoria 3168 Australia; 6 School of Pharmacy, University of Nottingham, Nottingham, NG7 2RD UK

## Abstract

Preventing biological contamination (biofouling) is key to successful development of novel surface and nanoparticle-based technologies in the manufacturing industry and biomedicine. Protein adsorption is a crucial mediator of the interactions at the bio – nano -materials interface but is not well understood. Although general, empirical rules have been developed to guide the design of protein-resistant surface coatings, they are still largely *qualitative*. Herein we demonstrate that this knowledge gap can be addressed by using machine learning approaches to extract *quantitative* relationships between the material surface chemistry and the protein adsorption characteristics. We illustrate how robust linear and non-linear models can be constructed to accurately predict the percentage of protein adsorbed onto these surfaces using lysozyme or fibrinogen as prototype common contaminants. Our computational models could recapitulate the adsorption of proteins on functionalised surfaces in a test set with an *r*^2^ of 0.82 and standard error of prediction of 13%. Using the same data set that enabled the development of the Whitesides rules, we discovered an extension to the original rules. We describe a workflow that can be applied to large, consistently obtained data sets covering a broad range of surface functional groups and protein types.

## Introduction

The behaviour of proteins on surfaces is of critical importance in a wide range of applications, particularly medical applications of nanomaterials^1^, biomedical implants, artificial tissue scaffolds or industrial applications where surfaces are compromised when exposed to microbial or other biological contaminants^2–8^. Protein adsorption at solid and liquid interfaces is a common but very complex phenomenon that is not well understood despite over four decades of research^2,3^. This paucity of mechanistic information on protein adsorption limits the rational design of the next generation of bioinert materials.

Poly(ethylene glycol) (PEG) derivatives have long been the gold-standard for antifouling materials, however, there are still a number of issues with these material^9^. PEG can be oxidised into non-biodegradable products whose impact on the body is currently unknown. Furthermore, in some circumstances, repeat exposure to PEGylated particles through multiple injections results in significant decrease in blood circulation time, limiting the efficacy of PEG functionalised particles^10–12^. A host of surface functionalizations, a wide range of zwitterionic, hydroxyl acrylate, oxazoline, vinylpyrrolidone, and glycerol polymers, peptides, and peptoids, have been used to block protein adsorption across a range of applications^13^, with varying degrees of success. Regardless of the system employed, the complex underlying mechanisms for, and influence of various surface chemistries on, protein adsorption are poorly understood.

**Figure 1:**
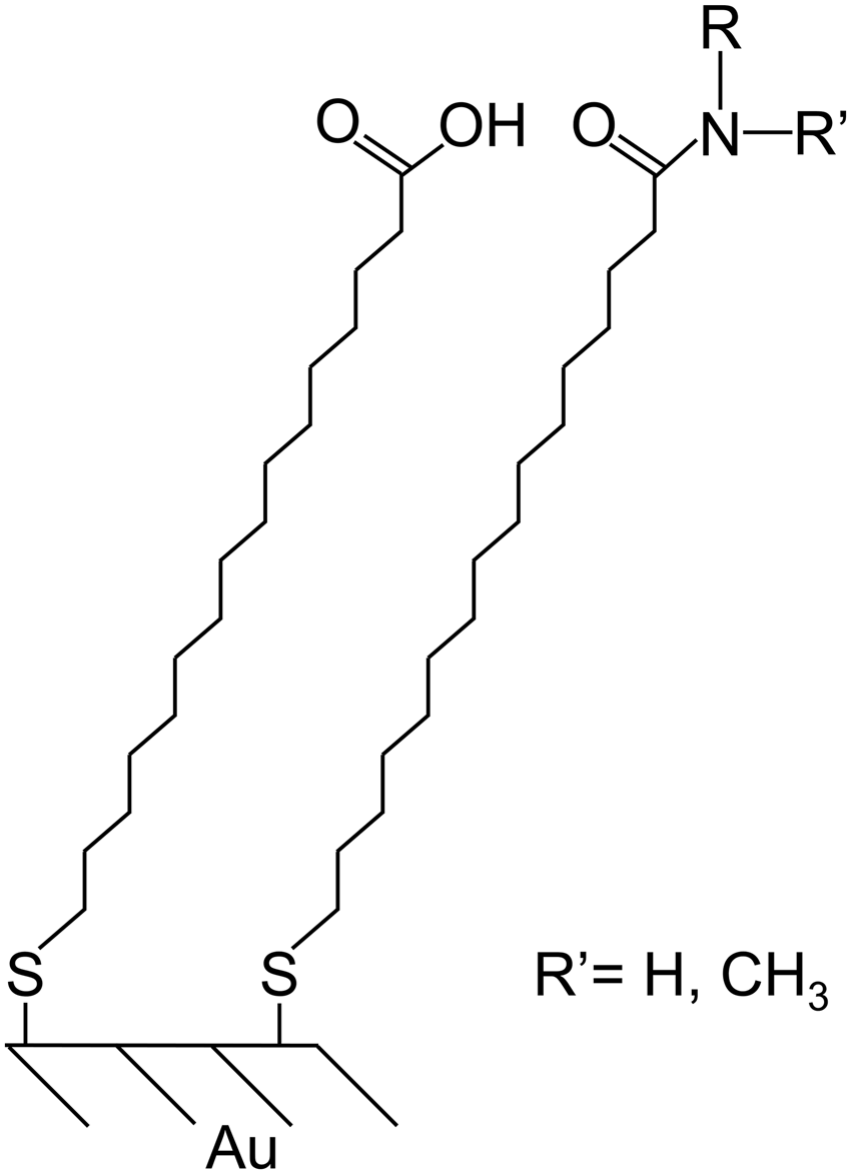
Chemical structure of the self-assembled monolayers (SAMs).

Among the general, empirical rules that have been proposed to aid the design of protein repellent surfaces, the “Whitesides rules” are arguably the most widely used. They arose from a systematic study of the protein adsorption capacity of 48 types of self-assembled monolayers (SAMs)^14,15^. These were prepared by the reaction of an amine HNR’R with a SAM that displays interchain carboxylic anhydrides on its surface, and their structure is shown in Fig. 1. Table 1 summarizes the compositions of the 48 SAMS, identifying the diversity of structures and physicochemical properties within this set of materials.

**Table 1:** The chemical structure of –R of the self-assembled monolayers (SAMs)^15^.

Entry	R	Entry	R	Entry	R	Entry	R
1	H_2_N(CH_2_)_10_CH_3_	13		25	H_2_N(Gly)_3_N(CH_3_)_2_	37	
2	H_2_NCH_2_(CF_2_)_6_CF_3_	14		26	H(CH_3_)N(Sar)_1_N(CH_3_)_2_	38	
3		15	H_2_N(CH_2_CH_2_O)_2_CH_2_CH_2_NH_2_	27	H(CH_3_)N(Sar)_3_N(CH_3_)_2_	39	HN(CH_2_CH_2_CN)_2_
4		16		28	H(CH_3_)N(Sar)_4_N(CH_3_)_2_	40	HN(CH_2_CN)_2_
5	H_2_NCH_2_CH_2_OCH_3_	17		29	H(CH_3_)N(Sar)_5_N(CH_3_)_2_	41	H_2_NCH_2_CH_2_CN
6	H_2_NCH_2_CH_2_OH	18	HN(CH_3_)_2_	30		42	
7	HN(CH_2_CH_2_OCH_3_)_2_	19		31		43	
8	H_2_N(CH_2_CH_2_O)_3_CH_3_	20		32		44	H_2_NC(CH_2_CH_2_CH_2_OH)_3_
9	H_2_N(CH_2_CH_2_O)_3_H	21		33		45	
10	H_2_N(CH_2_CH_2_O)_6_CH_3_	22		34		46	H(CH_3_)NCH_2_CH(OCH_3_)_2_
11	H_2_N(CH_2_CH_2_O)_6_H	23		35		47	
12		24	H_2_N(Gly)_1_N(CH_3_)_2_	36		48	

According to “Whitesides’ rules”, protein resistant surfaces should have the following characteristics:

- Polar (hydrophilic) functional groups and hydrogen bond acceptor groups.
- No hydrogen bond donor groups or net charge.

Although the rules are qualitative, they have been used to develop many types of bioinert surfaces such as oligo-/poly(ethylene glycol)s, oligo-/polyglycerols, and zwitterionic polymers^16^. The performance of alkane-SAM based coatings has now been somewhat superseded by hydrogel coatings and in many cases, coatings without the characteristics proposed by Whitesides *et al*. still have good performance. Researchers currently construct non-fouling layers underneath bioactive signalling molecules, rarely relying on alkane-SAMs but, instead, on hydrogel layers grafted to or from the surface. Unfortunately, there is a paucity of published data for hydrogels or polymer brushes that is consistently generated using the same experimental and measurement conditions and large enough to train machine learning models. There is a clear need for consistent and standardised procedures for generating experimental data on the adsorption of proteins at functionalised interfaces that can be used to generate more widely applicable machine models to aid in the understanding and design of new, efficient antifouling materials. To the best of our knowledge, the only previously reported application of machine learning to model the adsorption of proteins on material surfaces considered the adsorption of fibrinogen on polyarylate and polymethacrylate surfaces and no general design rules for these esters were reported^17–23^. Hence, this study aims to demonstrate the usefulness of statistical and machine learning techniques to identify quantitative relationships between the diverse chemistry of the material surface and the protein adsorption characteristics. These surfaces were functionalised with esters, ethers, amines, amides, sugars, nitriles and other functional groups. We use the same experimental data set from which the “Whitesides rules” were derived to illustrate that the technique can mine the data to extract established design rules *quantitatively* as well as initiate new rules.

## Methods

Machine learning methods have been very successful in many areas of molecular design for generating robust, predictive models linking microscopic structure and macroscopic properties of materials^24^. They are supervised learning methods that can extract the complex structure–activity (property) relationships from reliable data sets of molecules or materials whose microscopic structures are well defined and their macroscopic properties of interest are measured. Quantitative structure–property relationship (QSPR) techniques have been applied successfully to a broad range of materials properties from physical, chemical, and biological to mechanical, electronic, and optical properties^24^. In this work, we used QSPR techniques to derive the relationships between the chemical structures and physicochemical protperties of SAMs and their protein adsorption profile.

The adsorption data, consisting of the percentage protein monolayer coverage on a mixed SAM (%ML), reported by Ostuni *et al*^15^. was used to train the models. Four functional groups, (sulfonate, phosphate, chloro and fluoro) were underrepresented in the data. Underrepresented features cannot be adequately captured by the models therefore SAMs containing these groups were excluded. The combined data set (176 data points) used to train the models pertained to adsorption of lysozyme and fibrinogen at 3 and 30 minutes exposure times. These prototype proteins were used because they have different properties such as size, shape, and pI. Fibrinogen is a large (340 kDa) tetrameric aggregate with a pI of 5.5. It readily adsorbs onto hydrophobic and charged surfaces. It is similar to the extracellular matrix protein fibronectin. Lysozyme is a small (MW15 kDa), ubiquitous model protein with a pI of 10.9. It is positively charged at physiological pH. Molecular descriptors (mathematically encoded properties of molecules) used in the models related to structure, partial charges, existence of particular molecular fragments or functional groups, the molecular graph, and atomic mass and were calculated using the Dragon software^25^. Indicator variables specifying the protein type (lysozyme or fibrinogen) and time scale (3 or 30 minutes) were also included as descriptors. The total size of the pool of descriptors was 67.

Data sets were divided into training (80%) and test (20%) sets using the k-means clustering algorithm. Only the training set was used to generate the models. The ability of the models to predict the protein adsorption on SAMs not included in the training set was validated using the test set. Two QSPR modeling methods were employed: sparse multiple linear regression with expectation maximization (MLREM) and non-linear Bayesian regularized artificial neural networks with Bayesian prior (BRANNGP)^26–28^. The neural networks consisted of input, hidden, and output layers. The number of nodes in the input layer was equal to the number of descriptors and the output layer had only one node corresponding to the protein adsorption value %ML. Two or three nodes in the hidden layer were found to be sufficient to build good models. It has been shown that increasing the number is unnecessary as the Bayesian regularization automatically controls the complexity of the models to optimize the test’s predictive capacity^29^.

The performance of the models was assessed using the coefficient of determination (*r*^2^), the standard error of estimation (SEE), and the standard error of prediction (SEP). *r*^2^ is the square of the correlation coefficient between the predicted and measured %ML. SEE and SEP are the root-mean-square values, adjusted for degrees of freedom, of the difference between the predicted and measured %ML for the training and test sets respectively. SEE and SEP are more robust estimates of the predictive ability of models because, unlike *r*^2^, they do not depend on the number of data points in the training set or the number of descriptors in the model^30^. Predictive, robust models have *r*^2^ values close to 1.0 and SEE and SEP values that are similar and close to the experimental error.

## Results

### Whitesides Rules

We were interested in the degree to which the elements of “Whitesides’ rules” can make *quantitative* predictions of protein adsorption behaviour for the adsorption data set. Because the charged groups were underrepresented and excluded from the modelling data set, only three factors from ‘Whitesides rules’ remain as the model inputs: hydrophilicity; number of hydrogen bond acceptors; number of hydrogen bond donors. The hydrophilicity was represented by the hydrophilic factor (Hy)^31^ calculated using the Dragon software. Indicator variables for protein type and time at which measurements were made were also required, leading to a model containing 5 input parameters.

**Figure 2:**
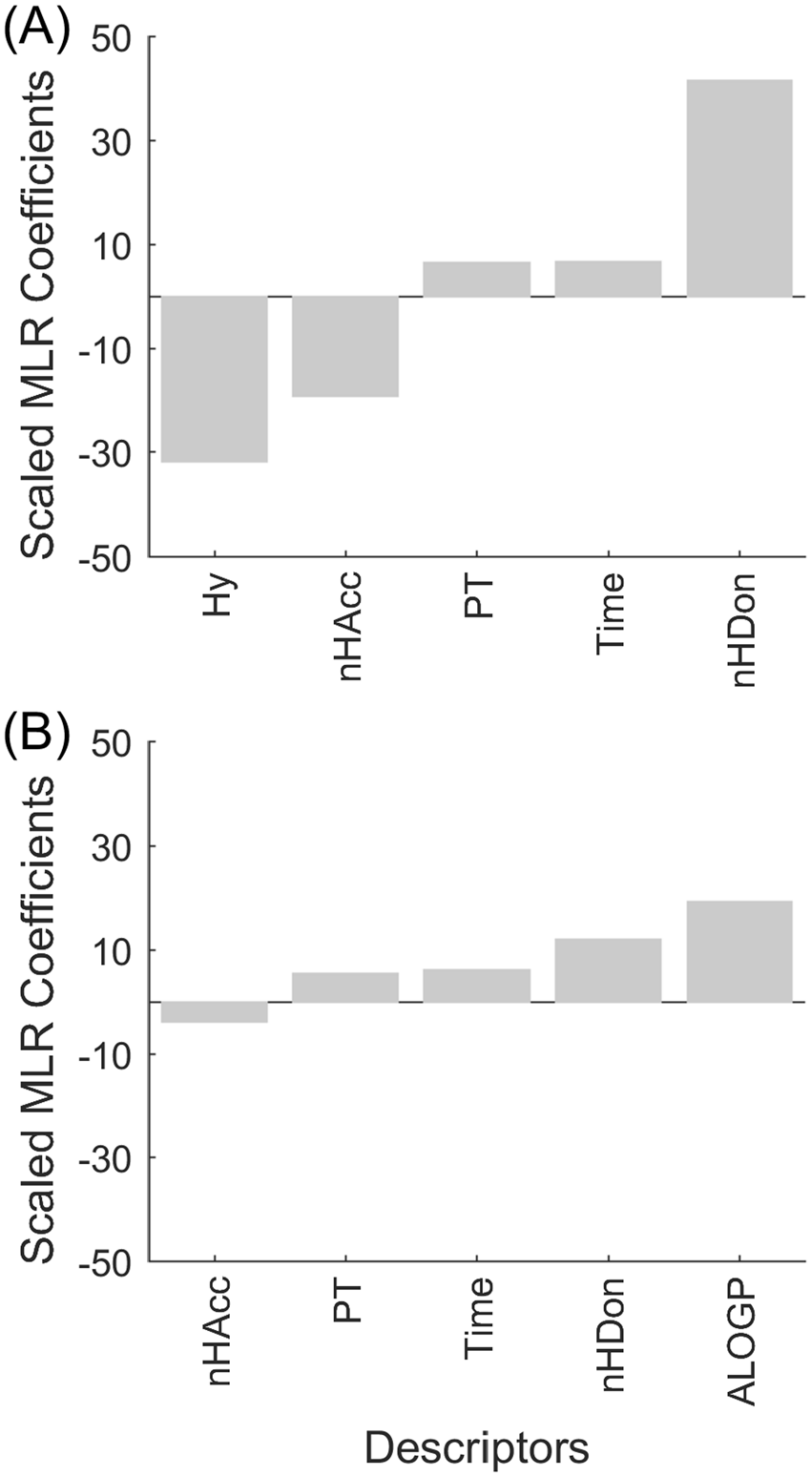
Scaled MLR coefficients for Whitesides rule descriptors to prevent protein adsorption. (**A**) Model using Hy parameter for hydrophilicity. (**B**) Model using AlogP for hydrophobicity.

Figure 2(A) shows the scaled (normalized) MLR coefficients generated for the model. These coefficients offer deeper understanding of the effect of each property on the degree of protein adsorption (%ML). A positive MLR coefficient indicates that the property promotes the adsorption of a protein onto the surface while a negative coefficient indicates that the property inhibits the adsorption. As Fig. 2(A) shows, the hydrophilicity (Hy) and the presence of hydrogen bond accepting functional groups (nHAcc) were associated with low protein adsorption. Larger number of hydrogen bond donor groups (nHDon) was associated with increased adsorption. These conclusions are in good agreement with the empirically derived rules. The three Whitesides’ rule properties have a much larger impact on protein adsorption than the protein type and or the time scale. Given that only two proteins were studied and the time points were reasonably short, this is not too surprising. However, the quantitative prediction ability of the model is poor, with a test set *r*^2^ value of 0.35 and standard error of prediction (SEP) of 24%. Replacement of the hydrophilic factor (Hy) with ALogP, the log octanol-water partition coefficient calculated using Ghose-Crippen-Viswanadhan method^32–34^, produces MLR coefficients again consistent with the Whiteside rules (Fig. 2(B)). The predictive power of this model improved slightly with the tests set *r*^2^ value rising to 0.54 and SEP dropping inconsequentially to 23%. However, both protein type and time made larger contributions to the model in this case, and the contribution of hydrogen bond acceptors was reduced. There is clearly a correlation between the donor/acceptor properties and the hydrophilicity/hydrophobicity properties, and logP values are influenced by the number of hydrogen bond donors.

A possible explanation for the relatively poor predictive power of these models is that the data set did not contain enough charged moieties to include them in the model, and the input parameters are all related to the ability of the functional ligands to interact with water and thus only reflect the hydration theory of protein repulsion^35^. To account for the steric repulsion theory of protein adsorption^36–38^ or a combination of both^2^, inclusion of parameters that reflect the dynamic character of ligands might improve the predictive power and design utility of the model.

### Comprehensive descriptor set

We computed a more comprehensive descriptor set using the Dragon package and employed both linear (MLREM) and non-linear (BRANNGP and BRANNLP) methods to make quantitative predictions of the protein adsorption behaviour. The initial pool of 67 Dragon descriptors which includes those capturing Whitesides original rules were pruned using MLREM sparse feature selection method^27,28^ to identify the most important descriptors that affect the protein adsorption. These approaches have been shown to be useful in carrying out sparse descriptor selection^39–42^. By tuning the sparsity of the MLREM progressively, the least informative descriptors were pruned out and the most relevant descriptors retained.

**Figure 3:**
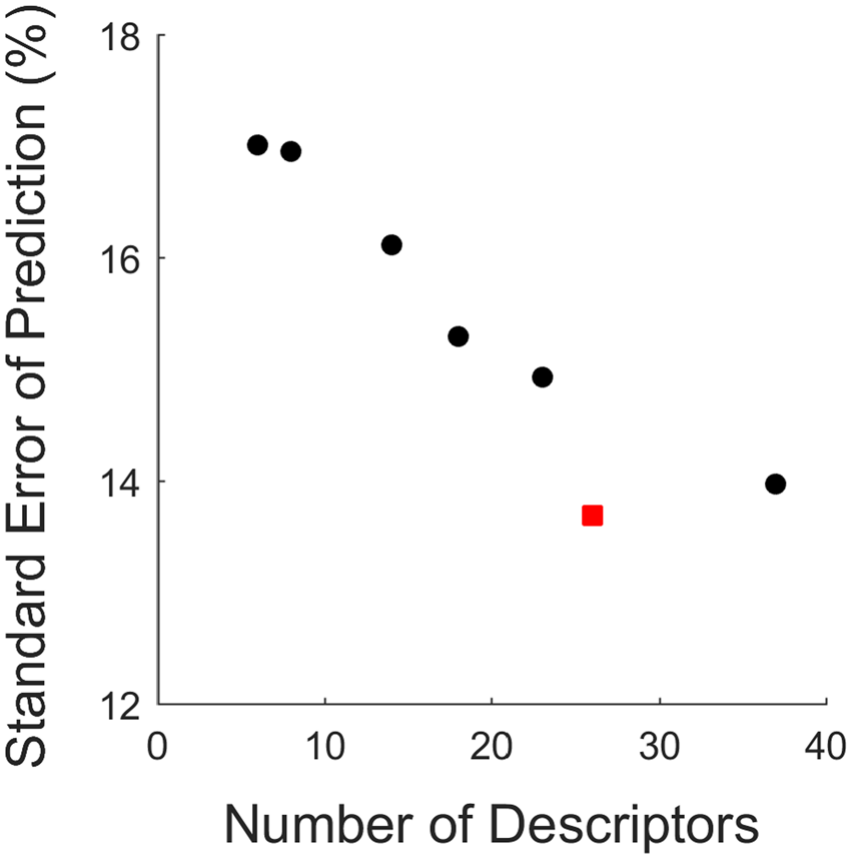
The dependence of the standard error of prediction (SEP) on the number of descriptors for models constructed using the MLREM approach to prune out irrelevant descriptors. The red data point indicates the best models with optimal sparsity.

**Figure 4:**
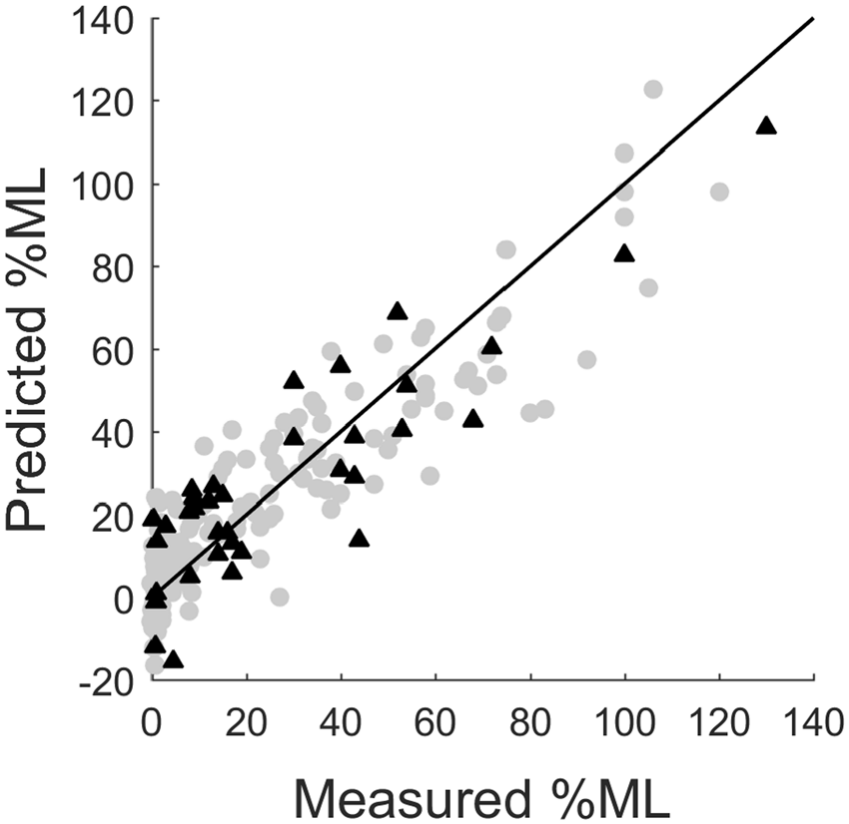
Prediction of the best MLREM model of percentage protein monolayer coverage on SAMs (%ML). Training set (grey circles) and test set (black triangles).

Figure 3 shows that by increasing the sparsity of the models and pruning out irrelevant descriptors, the predictive power of the models is enhanced and lower test set SEP values were obtained. When too many descriptors are removed, the performance of the models decreases and the SEP increases. The best models were those with the lowest SEP values and the lowest complexity (least number of descriptors). The performance of these models is summarized in Table 2 and Fig. 4. We also attempted to use different combinations of descriptors and the performance of the models using these descriptor sets is presented in Table S1.

**Table 2:** Statistics of the optimal linear and non-linear models of protein adsorption (fibrinogen and lysozyme) on different surfaces at 3 and 30 minutes. (*N*_*eff*_ is the number of effective weights (adjustable parameters) in the model).

Modelling technique	*N*_*eff*_	Training set		Test set	
		*r* ^2^	SEE [%]	*r* ^2^	SEP [%]
MLREM	27	0.81	13	0.78	14
BRANNGP^*^	28	0.82	12	0.76	14
BRANNGP^#^	35	0.84	10	0.79	14

^*^BRANNGP model built using the entire pool of 67 descriptors.

^#^BRANNGP model built using 25 descriptors selected by MLREM.

**Figure 5:**
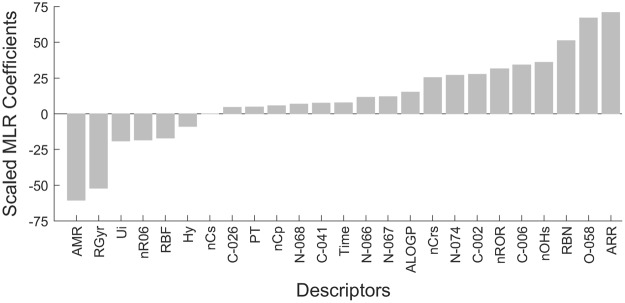
Scaled MLR coefficients of the most relevant descriptors selected from the pool 67 descriptors.

As can be seen in Table 2 and Fig. 3, the best models contain 25–28 descriptors or effective parameters and further pruning of descriptors result in a significant drop in predictive performance of the models. The linear and nonlinear models had equal ability to predict the %ML for SAMs in the test sets. This means that the relationship between the adsorption of protein on functionalized surfaces (%ML) and the descriptors is complex but largely linear. An examination of relevant structural descriptors selected by the models can provide some insight into the most significant factors that affect the adsorption process. The contribution of the most important descriptors is illustrated in Fig. 5 and the details of descriptors are listed in Table 3. When different sets of descriptors were used to construct the models, the contribution of these descriptors is presented in Fig. S1.

**Table 3:** The most relevant descriptors selected by MLREM and their contributions to the model predicting %ML. The descriptors are listed in the order of least negative to most positive.

Descriptor	Definition	Type	Contribution
**Negative**			
AMR	Ghose-Crippen molar refractivity	Continuous	−60
RGyr	radius of gyration (mass weighted)	Continuous	−52
Ui	unsaturation index	Continuous	−19
nR06	number of 6-membered rings	Integer	−19
RBF	rotatable bond fraction	Integer	−17
Hy	hydrophilic factor	Continuous	−9
**Positive**			
C-026	number of R–CX–R	Integer	5
ProteinType	protein type indicator	Integer	5
nCp	number of terminal primary C(sp3)	Integer	6
N-068	number of Al_3_-N fragments	Integer	7
C-041	number of X-C(=X)-X fragments	Integer	8
Time	time scale indicator	Integer	8
N-066	number of Al-NH2 fragments	Integer	11
N-067	number of Al_2_-NH fragments	Integer	12
ALOGP	Ghose-Crippen octanol-water partition coeff. (logP)	Continuous	15
nCrs	number of ring secondary C(sp3)	Integer	25
N-074	number of R≡N / R=N- fragments	Integer	27
C-002	number of CH_2_R_2_ fragments	Integer	28
nROR	number of ethers (aliphatic)	Integer	32
C-006	number of CH_2_RX fragments	Integer	34
nOHs	number of secondary alcohols	Integer	36
RBN	number of rotatable bonds	Integer	51
O-058	number of O=	Integer	67
ARR	aromatic ratio	Integer	71

### Positive contributors to adsorption

The examination of the positive descriptors and their associated scaled MLR values (Fig. 5) shows that 5 of the top 10 predictors of protein adsorption apply only to a specific group of ligands. These descriptors can therefore be classified as primary exclusion criteria and these types of chemistries should not be considered for inclusion in antifouling ligands. For example, both aromatic groups and nitriles show high protein adsorption and this is reflected in the presence of the descriptor ARR and N-074 as positive contributors to protein adsorption. The hydrogen bond donating secondary alcohols (nOHs) and NHR_2_ groups (N-066 and N-067) can be classified as promoting adsorption. The NHR_2_ descriptor must be taken with some caution as all ligands contain either one N-066 and N-067 based on the covalent attachment point.

### Negative contributors to adsorption

Four of the six descriptors that make negative contributors to the protein adsorption model are continuous descriptors that adopt non-zero values for all ligands. Molar refractivity (AMR), a measure of ligand size and polarizability^43^, has the largest negative impact on protein adsorption. The trend of decreasing adsorption with increasing substituent size is evident from observation of, for example, linear EG derivatives or substituted amide groups.

The final two continuous descriptors relate to the conformational flexibility of the ligands: radius of gyration (RGyr) and rotatable bond fraction (RBF). Both have a relatively strong correlation (>0.84) to the number of rotatable bonds (RBN) which was a positive contributor to protein adsorption. Given the respective scaled MLR coefficients the combination of these three parameters suggests that increased ligand conformational freedom deters protein adsorption.

The remaining two descriptors relate to specific ligand chemistry. Ten ligands contain aromatic rings (non-zero nR06 descriptors, number 6 membered rings), of these, 6 have applicable primary exclusion criteria, either aromatic content or secondary hydrogen bonds. Similarly, 23 ligands have non-zero unsaturation indices (Ui), 10 of which have defined primary exclusion criteria. 11 of the remaining 13 ligands are amino acids that exhibit decreasing adsorption with increasing number of repeating units, consistent with Ui being a negative contributor to adsorption.

### Reconciling QSPR predictions with Whitesides Rules and existing theories

Two theories exist regarding the underlying mechanism of protein resistance by SAM protected surfaces: steric repulsion and hydration theory. Steric repulsion rationalises protein adsorption resistance based on the conformational freedom of surface grafted ligands in good solvent conditions which present a high entropic penalty working against protein adsorption^37,38,44^. Hydration theory^35^ was developed to account for high density SAMs, where ligands would have restricted dynamics, yet showed resistance to protein adsorption. It has been reported that the capacity for functional ligands to coordinate water within the SAM layers and at the SAM/water interface is critical to limiting protein adsorption^11^. Hybrids of the two have been reported for PEG systems^45^ and recent MD simulations support the synergistic influence of these two factors in preventing protein adsorption^2^.

Ostensibly Whitesides Rules fall within the hydration theory of protein adsorption resistance as they only account for ligand chemistries related to the interaction of water with the functional ligand. Interestingly, there is a significant number of reported ligands that partially contradict these general rules and still possess protein adsorption resistance^16^. This can be explained by the fact that in the original derivation the rules were not accounting for the steric repulsion theory and the entropic contribution of the ligands to protein resistance, possibly amongst other contributing factors. Nevertheless, the rules, together with the data set from which they are derived, provide an excellent framework for refinement using the QSPR models via chemical identification along with systemic understanding to create improved models with greater predictive power.

**Figure 6:**
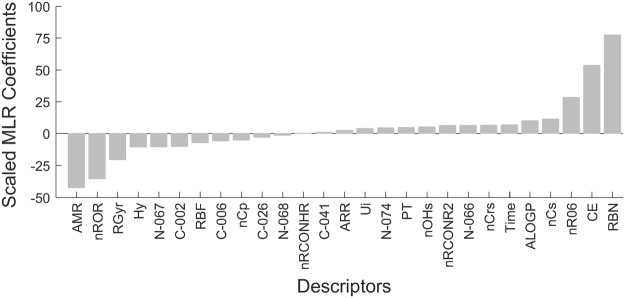
Scaled MLR coefficients of descriptors in the updated model.

**Figure 7:**
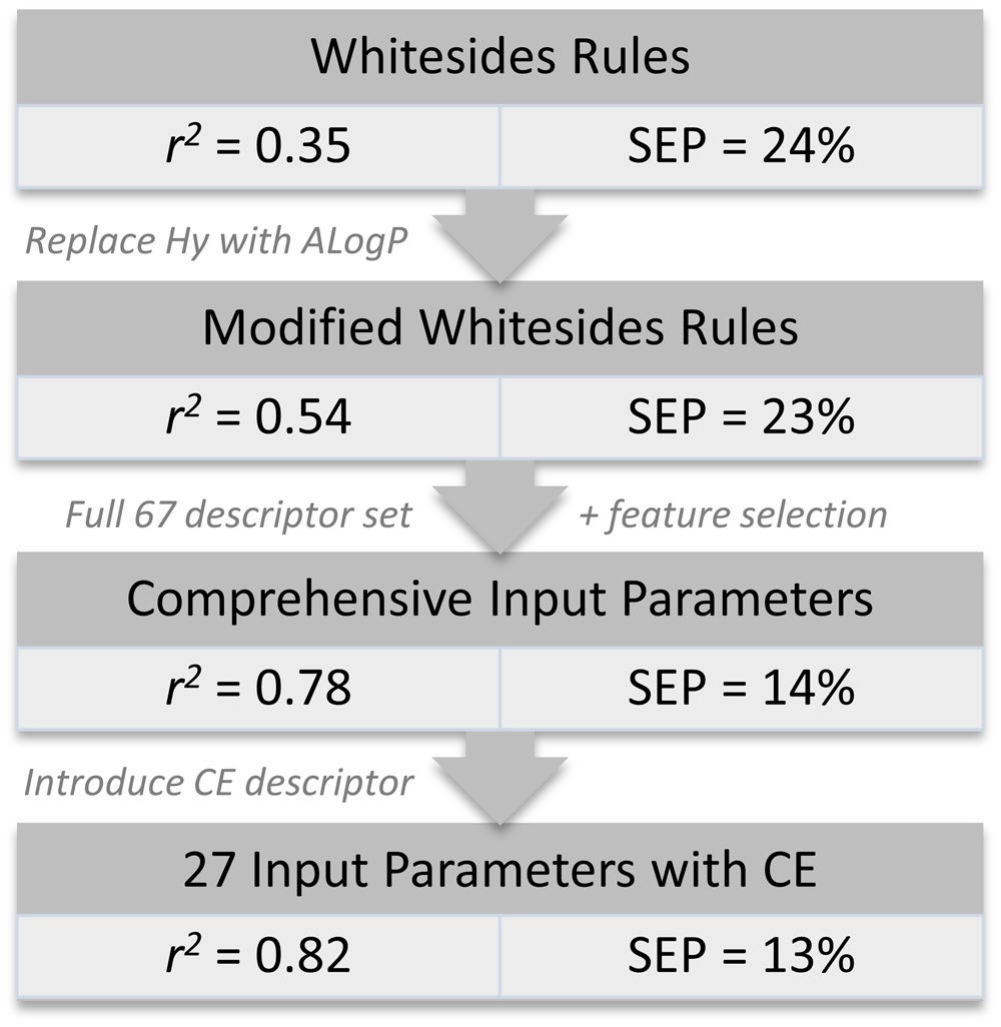
The workflow and the corresponding evolvement of the model performance.

When a broader range of properties captured by descriptors were added to the models, the quality of the prediction of protein adsorption improved markedly (test set *r*^2^ value of 0.78 and SEP of 14% compared to 0.35 and 23% and respectively). Lipophilic properties captured by the descriptors ALOGP, nCrs, C-002, C-006, ARR were strongly associated with high protein adsorption, consistent with widely reported findings^3^. Neither the total number of hydrogen bond donors (nHDon) nor acceptors (nHAcc) in general were identified as significant for predicting the extent of protein adsorption. The former was captured in the model using the comprehensive descriptor set by the number of secondary alcohols and (to a lesser extent) primary and secondary amines (N-066, N-067, nOHs) which did promote adsorption in line with the Whitesides rules. The models also predicted that the number of oxygens with double bond (O-058) and the total number of aliphatic ether (nROR) moieties (both hydrogen bond acceptors) contributed positively to adsorption. This is inconsistent with the Whitesides’ rules and, more surprisingly, the well-established role of polyethylene glycol (PEG) as the gold standard in preventing non-specific protein adsorption. To improve the models, we added an indicator variable to differentiate between the crown ether type structures which cause high protein adsorption rate and linear ethers (PEGs) leading to the low protein adsorption. We also replaced O-058 in the model with more specific descriptors for amides (nRCONH2, nRCONR2, and nRCONHR), and ketone (nRCOR) which were in the original pool of descriptors but got pruned out during the feature selection step. Although the newly obtained model only has slightly better performance with the test set *r*^2^ value of 0.82 and standard error of prediction of 13%, it is able to differentiate between the high and low adsorption polyethylene glycols as can be seen in the highly negative MLR coefficient for nROR and highly positive coefficient for the crown ether indicator in Fig. 6. The scaled MLR coefficients of descriptors in the obtained model using crown ether indicator are reported in Table S2 of the Supplementary Information. The workflow and the corresponding evolvement of the model performance are summarized in Fig. 7.

Modifications to the QSPR model are critical to aid in interpretation of the model results, particularly when the model identifies criteria with positive contributions from hydrogen bond acceptors that directly contradict both well-established theory and empirical results. The important role of molar refractivity, radius of gyration and rotatable bond fraction in promoting low attachment was maintained with the addition of the flag for crown ether (CE), see Figs 5 and 6. These parameters are associated with ligand dynamics, size and polarizability, factors that were not captured by Whitesides original rules. By adding the first two descriptors (AMR and RGyr) to the model using the original descriptors from Whitesides rules (Hy, nHDon, nHAcc), the test set r^2^ value of the model increases significantly from 0.35 to 0.53 and the SEP dropped from 23% to 20%. Moreover, since an increased predictive power was previously observed when ALogP replaced Hy in the model, we attempted to build a model using the set of AMR, RGyr, ALogP, nHDon and nHAcc. As expected, when Hy is replaced by ALogP, the performance of the model improved further, with the test set r^2^ value of 0.56 and SEP of 17%.

Using the same strategy, we constructed models using different combinations of descriptors and provided the details in Table S1 and Fig. S2 of the Supporting Information. The observed importance of the dynamic factors (AMR and RGyr) suggests a synergistic effect between hydration and steric mechanisms in the ability of functional SAM to resist protein adsorption. We, therefore, propose that Whitesides’ original rules be extended as follows. For self-assembled functional ligands to resist protein adsorption they should comprise:

- polar (hydrophilic) functional groups and hydrogen bond acceptor groups.
- no hydrogen bond donor groups or net charge.
- relatively *large, conformationally mobile and polarizable functional groups*.

Obviously, the additional properties (size, flexibility and polarizability) are highly related to the surface coating density which has been shown to play an important role in the antifouling ability of materials^10,46–50^. In the original work, the grafting density was not reported and therefore this information was not included in our models. The inclusion of grafting density or defects in the layers as inputs might improve the predictability of the models.

### Limitations and Improvements

An important limitation is the under-representation of charged functional groups in the model training set. Clearly, a larger and more diverse data set will improve the predictions of the models and strengthen the validity of the empirical design rules the Whitesides group developed and we have extended. It must be noted that the predictive power of the model is within experimental error despite a number of limitations in the original data set used for this initial proof of concept utilising only calculable data for each molecule. However, the model can be further improved if the limitations are addressed. For example, while in the current model no consideration is given to the mode of the ligand attachment to the surface, it can be assumed that this region will have lesser impact on adsorption behaviour and can be accounted for in future models. However, the constraints associated with ligand attachment can have an influence on the overall dynamics of the ligand, reducing the RGyr, and, therefore, inclusion of relevant descriptors in future predictions should be considered. There are also parameters external to the ligand chemistry which will influence protein adsorption behaviour. For example, here, all ligands were treated equally with regards to grafting density (surface coverage) which was unreported in the original work, while it is known that the grafting density can influence protein adsorption behaviour^10^. Furthermore, in the original work, the authors presented data relating protein adsorption behaviour to advancing water contact angle in cyclooctane and found no correlation. While water contact angle is not a good predictor of protein adsorption in isolation it will likely be very useful when considered as one of numerous factors facilitating or preventing adsorption. Inclusion of these experimentally measurable (non-computed) properties will likely increase the predictive power of the model as it will more accurately reflect the physical system. Lastly, it has been shown that the interfacial bound water and its distinct properties influence the protein adsorption profile^51^. Hence the addition of parameters characterizing the hydration layer structure of the materials as input descriptors may improve the model performance.

Only two proteins were presented in the original data set available and it would clearly be useful to measure the attachment of a wider range of proteins with more diverse properties (size, lipophilicity, shape etc.) To this end, in our QSPR models, the protein type flag was treated as binary. In every model protein type was found to be a positive predictor of protein adsorption indicating that fibrinogen (flagged as 1) had a higher adsorption propensity than lysozyme (flagged as 0). While the use of a binary flag was sufficient for the proof of concept presented here, more comprehensive parameters to describe the adsorbing proteins should be considered in future QSPR models. A method for encoding the nature of more diverse types of proteins will most likely improve the models and the rules. Also, proteins are not passive in the adsorption process. A partial list of properties that might be considered which have previously been reported to play a role in protein adsorption are size, surface composition^52^, conformational flexibility (conformational ‘hardness’ or ‘softness’)^53^, surface activity or organisation capacity (i.e. clusterin^54,55^; hydrophobin^56^; and adhesins^57^).

A final challenging aspect of understanding the various influences at work remains the lack of consistency in available experimental data. A wide range of protein properties, including the amount of adsorbed protein, have been reported under different conditions. The efficacy of the models presented, based on an older and somewhat limited data set, suggests that with a standardised procedure for generating experimental data on the adsorption of proteins at functionalised interfaces QSPR models could greatly aid in the understanding and design in this space.

## Conclusions

Understanding the effect of surface chemistry on protein adsorption is critical for the design of novel bioinert materials. We have shown how computational modelling using machine learning algorithms can generate a quantitative relationship between surface chemistry and protein adsorption. The models elucidate design concepts through the model weights of over 20 physical/nonphysical parameters. Such concepts can be useful for designing protein resistant as well as protein attracting surfaces. The models also highlighted the challenge of balancing related properties which deter or promote protein adsorption and supported the notion of synergy between hydration and steric effects in preventing adsorption. The model is capable of reliably predicting the degree of protein adsorbed on SAMs (within the applicability domain of the models) for new surface chemistries. Therefore, the machine learning based predictions are demonstrated to be useful to identify surfaces with the best performance for synthesis and fabrication. However, this requires more robust data sets with good quality molecular level data characterising the interface to which proteins are exposed under practically relevant conditions to allow QSPR models to become more widely applied in the diverse range of technologies reliant on mediation of protein adsorption.

## Electronic supplementary material

Supplementary Information

## Data Availability

The raw/processed data required to reproduce these findings are available to download from https://drive.google.com/drive/u/0/folders/1o4noYh7dXnYg113kaJdLOjseuTTMlNQf.
