# Supplementary material for: Quantitative design rules for protein-resistant surface coatings using machine learning
Source: Sci Rep. 2019 Jan 22;9:265. doi: 10.1038/s41598-018-36597-5 (PMC6342937; doi:10.1038/s41598-018-36597-5)
Supplement: Supplementary file 1 — Supplementary Information [file 41598_2018_36597_MOESM1_ESM.pdf]

Quantitative design rules for protein-resistant surface coatings using machine learning

Tu C. Le[1,*], Matthew Penna[1,2], David A. Winkler[3,4,5,6], Irene Yarovsky[1,2,*]

[1]School of Engineering, RMIT University, GPO Box 2476, Melbourne, Victoria, 3001, Australia

[2]ARC Research Hub for Australian Steel Manufacturing, Australia

[3]Monash Institute of Pharmaceutical Sciences, Monash University, Parkville, Victoria, 3052, Australia

[4]Latrobe Institute for Molecular Science, La Trobe University, Bundoora, Victoria 3084, Australia

[5]School of Chemical and Physical Sciences, Flinders University, Bedford Park, South Australia, 5042, Australia

[6]School of Pharmacy, University of Nottingham, Nottingham NG7 2RD, UK

*Corresponding authors:

Email: Tu.Le@rmit.edu.au. Phone: +61 9925 2216

Email: Irene.Yarovsky@rmit.edu.au. Phone: +61 9925 2571

Table S1. Statistics of the linear models for protein (fibrinogen and lysozyme) adsorption on different surfaces at 3 and 30 minutes using different sets of descriptors.

Descriptors used (ProteinType and Time are included in all models)	Training set		Test set	
	r^2	SEE [%]	r^2	SEP [%]
(a) nHAcc, nHDon, Hy	0.39	23	0.35	24
(b) nHAcc, nHDon, ALOGP	0.62	18	0.54	23
(c) nHAcc, nHDon, Hy, RGyr	0.40	23	0.32	25
(d) nHAcc, nHDon, ALOGP, RGyr	0.62	19	0.60	18
(e) nHAcc, nHDon, Hy, Rgyr, AMR	0.51	21	0.53	20
(f) nHAcc, nHDon, ALOGP, RGyr, AMR	0.63	19	0.56	17
(g) nHAcc, nHDon, Hy, RBF	0.43	22	0.31	25
(h) nHAcc, nHDon, ALOGP, RBF	0.64	17	0.55	23
(i) nHAcc, nHDon, Hy, RGyr, RBF	0.43	22	0.36	24
(j) nHAcc, nHDon, ALOGP, RGyr, RBF	0.62	19	0.60	18
(k) nHAcc, nHDon, Hy, ALOGP	0.59	20	0.80	14
(l) nHAcc, nHDon, Hy, ALOGP, RGyr	0.60	19	0.78	14
(m) nHAcc, nHDon, Hy, ALOGP, RGyr, AMR	0.60	19	0.73	14
(n) nHAcc, nHDon, Hy, ALOGP, RGyr, AMR, RBF	0.61	19	0.73	15
(o) nHAcc, nHDon, Hy, CE	0.39	24	0.41	21
(p) nHAcc, nHDon, Hy, CE, RGyr	0.37	24	0.47	21
(q) nHAcc, nHDon, ALOGP, CE, RGyr	0.59	20	0.75	14
(r) nHAcc, nHDon, Hy, ALOGP, CE	0.59	20	0.79	14
(s) nHAcc, nHDon, Hy, ALOGP, CE, RGyr	0.61	19	0.78	14
(t) nHAcc, nHDon, Hy, ALOGP, CE, RGyr, AMR	0.61	19	0.73	15
(u) nHAcc, nHDon, Hy, ALOGP, CE, RGyr, AMR, RBF	0.61	19	0.73	14

Figure S1. Scaled MLR coefficients of models built using different sets of descriptors.

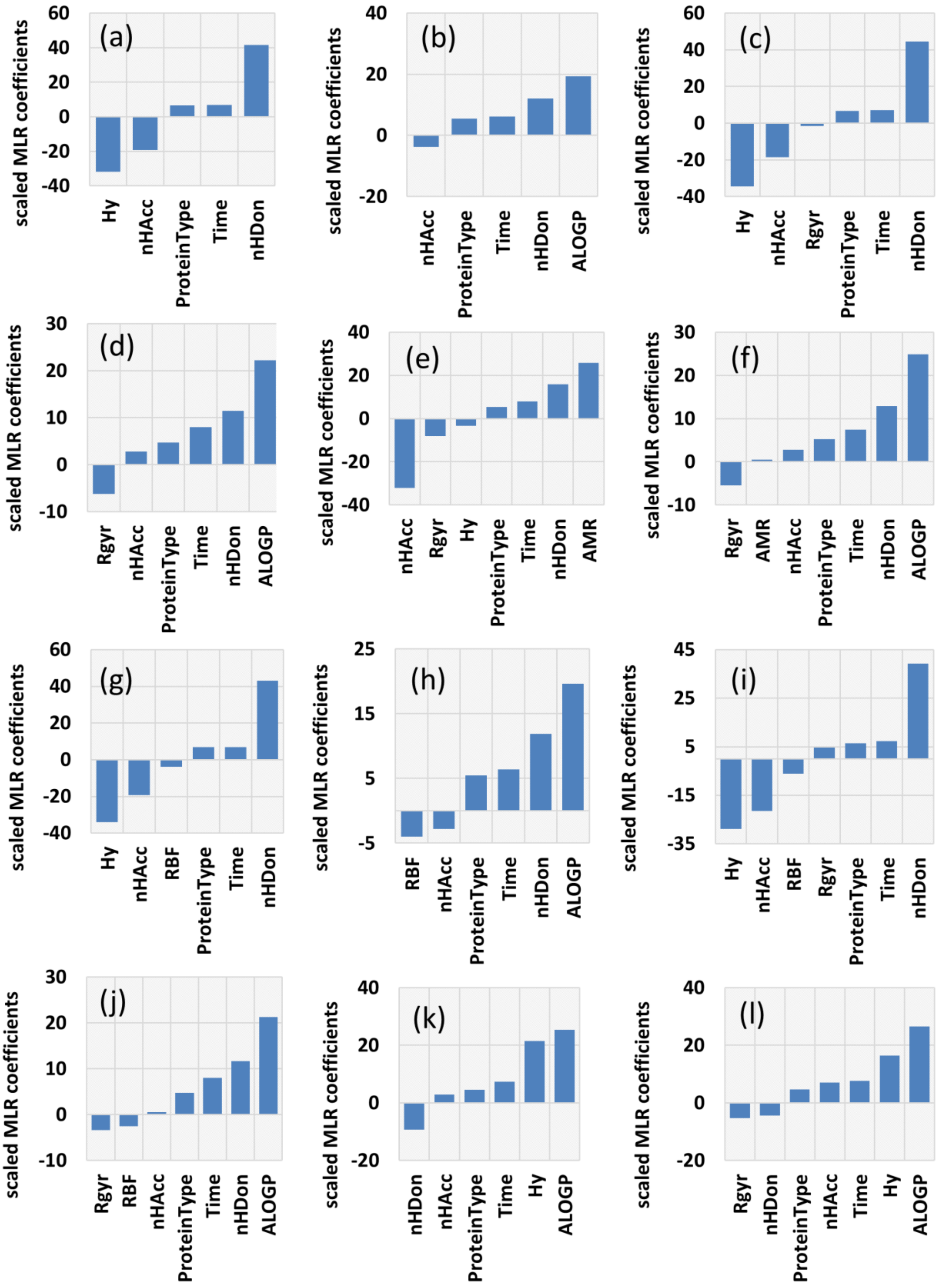

Table S2. Scaled MLR coefficients of descriptors in the obtained model that uses crown ether indicator and O-058 replacement descriptors (figure 6).

Descriptor	Coefficient
AMR	-42.483373
nROR	-35.749337
RGyr	-20.73896
Hy	-10.742455
N-067	-10.605714
C-002	-10.291102
RBF	-7.343363
C-006	-5.915241
nCp	-5.271716
C-026	-3.077771
N-068	-1.492334
nRCONHR	0.255736
C-041	0.975001
ARR	2.500026
Ui	3.942591
N-074	4.477056
ProteinType	4.771819
nOHs	5.217762
nRCONR2	6.355406
N-066	6.415772
nCrs	6.530366
Time	6.873053
ALOGP	10.084223
nCs	11.396105
nR06	28.435337
CE	53.687128
RBN	77.374377
